# An Axiology of Information Security for Futuristic Neuroprostheses: Upholding Human Values in the Context of Technological Posthumanization

**DOI:** 10.3389/fnins.2017.00605

**Published:** 2017-11-07

**Authors:** Matthew E. Gladden

**Affiliations:** Institute of Computer Science, Polish Academy of Sciences, Warsaw, Poland

**Keywords:** information security, CIA triad, neuroprostheses, human-computer interaction, technological posthumanization, philosophy of technology, axiology

## Abstract

Previous works exploring the challenges of ensuring information security for neuroprosthetic devices and their users have typically built on the traditional InfoSec concept of the “CIA Triad” of confidentiality, integrity, and availability. However, we argue that the CIA Triad provides an increasingly inadequate foundation for envisioning information security for neuroprostheses, insofar as it presumes that (1) any computational systems to be secured are merely instruments for expressing their human users' agency, and (2) computing devices are conceptually and practically separable from their users. Drawing on contemporary philosophy of technology and philosophical and critical posthumanist analysis, we contend that futuristic neuroprostheses could conceivably violate these basic InfoSec presumptions, insofar as (1) they may alter or supplant their users' biological agency rather than simply supporting it, and (2) they may structurally and functionally fuse with their users to create qualitatively novel “posthumanized” human-machine systems that cannot be secured as though they were conventional computing devices. Simultaneously, it is noted that many of the goals that have been proposed for future neuroprostheses by InfoSec researchers (e.g., relating to aesthetics, human dignity, authenticity, free will, and cultural sensitivity) fall outside the scope of InfoSec as it has historically been understood and touch on a wide range of ethical, aesthetic, physical, metaphysical, psychological, economic, and social values. We suggest that the field of axiology can provide useful frameworks for more effectively identifying, analyzing, and prioritizing such diverse types of values and goods that can (and should) be pursued through InfoSec practices for futuristic neuroprostheses.

## Introduction

### The unique information security needs of neuroprostheses

Generic information security (InfoSec) mechanisms like antivirus software and file encryption tools that are useful for safeguarding desktop computers are often inapplicable or unsound for use in securing complex medical technologies. Researchers have thus sought to develop more tailored InfoSec practices for medical information systems (Bergamasco et al., 2001; Freudenthal et al., 2007; Clark and Fu, 2012) and implantable medical devices (Denning et al., 2008, 2010; Halperin et al., 2008; Rasmussen et al., 2009; Hansen and Hansen, 2010; Schechter, 2010; Hei and Du, 2011; Cho and Lee, 2012; Rotter and Gasson, 2012; Zheng et al., 2014). Similarly, researchers have sought to identify unique InfoSec challenges posed by neuroprosthetic devices, which—due to their integration with the human nervous system—require specialized InfoSec mechanisms that are irrelevant for other types of IMDs (Denning et al., 2009; Bonaci, 2015; Bonaci et al., 2015a,b).

### The difficulty of formally defining the goals of information security

Such research only infrequently explores the question of exactly what is meant by “information security.” How would one recognize whether efforts to achieve it are succeeding or failing? The majority of texts noted above do not explicitly endorse any existing InfoSec frameworks that formally define goals for information security. Those texts that do explicitly base their analysis on an established definition of information security (Denning et al., 2008, 2009; Halperin et al., 2008; Bonaci, 2015; Bonaci et al., 2015a,b) opt for the classic “CIA Triad,” which was developed in the 1970s and establishes *confidentiality, integrity*, and *availability* as the three overarching aims of information security. Here “confidentiality” means that disclosure of information is successfully limited to authorized parties, “integrity” means that information is protected from degradation or illicit manipulation, and “availability” means that information can be accessed by authorized users in a timely and reliable manner (Samonas and Coss, 2014).

Selecting the CIA Triad as a conceptual foundation is reasonable, as it is the simplest and best-known InfoSec framework that explicates information security's goals. However, while its value as a pedagogical tool for introducing basic InfoSec principles remains unsurpassed, within the field of InfoSec the CIA Triad's limitations as an instrument for designing comprehensive security practices have gradually become apparent. InfoSec researchers have thus proposed more nuanced frameworks to capture additional aspects of information security (Samonas and Coss, 2014). For example, the Parkerian Hexad adds the goals of *possession, authenticity*, and *utility* (Parker, 2002, 2010), while goals proposed by others include *accuracy, completeness, consistency, non-repudiation, relevance*, and *timeliness* (Dardick, 2010).

### Ways in which futuristic neuroprostheses challenge traditional InfoSec assumptions

In themselves, such developments suggest that neuroprosthetics researchers should no longer presume that the CIA Triad offers an appropriate starting point for exploring InfoSec for neuroprostheses. However, we would suggest two further reasons why the Triad provides an obsolete (and potentially even harmful) basis for analyzing InfoSec for futuristic neuroprostheses. Namely, some future neuroprostheses can be expected to violate the Triad's implicit assumptions that (1) computational systems to be secured are ultimately nothing more than instruments for expressing the agency of their human users, and (2) computing devices are conceptually and practically separable from their human users. Insofar as future neuroprostheses break those conditions, any InfoSec regimes designed for them on the basis of the CIA Triad may lack some security mechanisms needed to fully protect devices and their users while simultaneously implementing other mechanisms that can prove detrimental. Below we consider these points further.

## Securing futuristic neuroprostheses that are more than simply tools

### Classical InfoSec frameworks' instrumental approach

The distinguishing feature of an “agent” is its possession of some degree of autonomous decision-making and action. Both human beings and artificial computing devices constitute agents, in different ways. The “strong” form of *biological agency* possessed by human beings is a complex amalgam of phenomena including conscious awareness; imagination; volition; conscience; rational decision-making influenced by emotion, instinct, and cognitive biases; and the embodiment of each mind within a unique physical form. This differs greatly from the “weak” form of *artificial agency* possessed by contemporary electronic computers, which possess a more limited and straightforward ability to process data and select a course of action without ongoing direct human control (Wooldridge and Jennings, 1995; Lind, 2001; Friedenberg, 2008; Fleischmann, 2009).

The CIA Triad arose in the 1970s as a practical aid for securing electronic computers that were processing increasingly sensitive and critical data. Built into the Triad is the historical assumption that an information system to be secured is not a biological agent but an expendable tool whose value subsists in the fact that it helps human users more effectively exercise their own biological agency by aiding them to process information, make decisions, and act for their own chosen ends. From that instrumental perspective, ensuring the confidentiality, integrity, and availability of information contained in a computer was considered sufficient to ensure the computer's adequate functioning as a tool for human use.

Most contemporary neuroprostheses are governed by computers constituting straightforward artificial agents, and the neuroprostheses themselves fill recognizable instrumental roles: for example, cochlear implants, retinal prostheses, and robotic prosthetic limbs allow human beings with certain medical conditions to perceive and manipulate their environment more effectively, while devices capable of detecting and interpreting a user's thoughts allow paralyzed but conscious patients to express their wishes (Merkel et al., 2007; McGee, 2008; Edlinger et al., 2011; Gasson et al., 2012; Lebedev, 2014).

### Emerging challenges to the instrumental vision of neuroprostheses

The instrumental vision of technology presented above accepts the “neutrality thesis” that technological devices are created by human designers through the exercise of “instrumental rationality” and exist merely as passive tools that can be applied equally for either good or bad purposes. However, that view has been vigorously challenged as simplistic or wholly incorrect from various philosophical perspectives by thinkers like Heidegger, Marcuse, Ellul, Habermas, Virilio, Latour, and Fukuyama (Ellul, 1964; Habermas, 1970; Heidegger, 1977; Latour, 1996; Virilio, 1999; Marcuse, 2001, 2011; Fukuyama, 2002; Franssen et al., 2015).

Moreover, InfoSec's traditional instrumental model is expected to increasingly be undermined at the technological level by unconventional information systems like DNA-based and biological computers, physical (e.g., memristive) neural networks, nanorobotic swarms, evolvable software, self-improving robots, and hypothesized future forms of artificial general intelligence whose exercise of agency cannot necessarily be “programmed” or directly controlled by human beings for their own purposes (Friedenberg, 2008; Pearce, 2012; Yampolskiy and Fox, 2012; Gunkel, 2017). Insofar as future neuroprostheses incorporate such technologies, they may be less likely to simply support their hosts' biological agency; they might instead conceivably impair, override, transform, or replace it. This might be encountered, for example, with neuroprostheses that are controlled by computers possessing human-like cognitive abilities or are composed of biological components possessing their own biological agency distinct from that of their users (Rutten et al., 2007; Stieglitz, 2007; Gladden, 2016b).

Neuroprostheses' complex relationship to their users' agency is already revealed by existing devices. For example, it has been anecdotally noted that some users of deep brain stimulation implants report that their implants have strengthened their sense of autonomy and human agency: by treating disorders that had robbed them of motor control over their bodies, such devices have allowed their users to feel like “themselves” again for the first time in years. However, an opposite reaction has been anecdotally observed among other DBS users, who report that the devices undermine their sense of possessing full human agency, as they fear they can never entirely know which of their thoughts are truly “their own” and which might be generated by their implants (Kraemer, 2011; Van den Berg, 2012).

### Futuristic neuroprostheses' intimate and ambivalent relationship with human agency

Futuristic neuroprostheses' relationship to their users' agency is expected to be even more fraught. For example, if researchers build on technologies already successfully tested in mice (Han et al., 2009; Josselyn, 2010; Ramirez et al., 2013) to develop neuroprostheses capable of interpreting, creating, altering, or erasing human beings' long-term memories, such devices might conceivably be used not only to treat phobias or aid with recovery from traumatic experiences (thereby enhancing patients' agency) but to alter or suppress memories of valued relationships, knowledge of moral principles, or the contents of firm decisions that an individual has already made—thereby impairing users' agency and replacing their judgment with that of the neuroprostheses' operators (Denning et al., 2009; Bonaci et al., 2015b).

The danger that neuroprostheses may not support their users' biological agency becomes more acute when considering the expected expansion of neuroprosthetics into the realm of human enhancement (Merkel et al., 2007; Gasson, 2008; McGee, 2008; Gasson et al., 2012). Future neuroprostheses may not be supplied by healthcare institutions interested solely in their patients' wellbeing but by military organizations deploying neuroprostheses to create more lethal augmented soldiers (Coker, 2004; Moreno, 2004; Kourany, 2014; Krishnan, 2015) or profit-oriented electronics firms seeking to offer computer gamers more immersive, thrilling, and potentially addictive VR experiences (Heidt, 1999; Kierkegaard, 2010; Scherer et al., 2012; Griffiths, 2017; Loup-Escande et al., 2017). It thus cannot be presumed—as the CIA Triad historically does—that enhancing the confidentiality, integrity, and availability of a device is equivalent to supporting the biological agency of its user. If ensuring such users' wellbeing is taken to be an important InfoSec aim, frameworks other than the CIA Triad will be needed to advance that goal.

## Human-machine integration: the need to secure the biocybernetic whole

### Historical InfoSec assumptions that computing devices are separable from their users

Also implicit in the CIA Triad's goals is an understanding that information to be secured is contained in some artificial information system other than a human mind, like a web server or smartphone. InfoSec does address dangers like social engineering attacks that target human users; however, at a theoretical level the CIA Triad largely presumes that computing devices are structurally and operationally separable from their human users (Samonas and Coss, 2014). When the CIA Triad is employed to design protections for a conventional computer, it may thus yield mechanisms like anti-tamper casings, file backup systems, and antivirus software designed to secure the computer *as a device*, independently of whoever uses it.

### The neuroprosthetic device and its user as elements of a larger biocybernetic system

It is expected, however, that future neuroprostheses may become structurally merged with their users' biological components and functionally integrated into their cognitive processes in powerful and intimate ways. Transdisciplinary research into futuristic neuroprostheses employing the tools of critical and philosophical posthumanism suggests that such devices may fuse with their human users through a process of “technological posthumanization” to create a qualitatively novel whole that is no longer simply a machine or a human being but a synthesis of the two possessing its own unique status (Hayles, 1999; Gray, 2002; Anderson, 2003; Clark, 2004; Herbrechter, 2013; Lilley, 2013; Naufel, 2013; Roden, 2014; Sandberg, 2014; Gladden, 2017). However, such analyses of the processes of “cyborgization” have had little impact on InfoSec, whose instrumental and technical perspective largely still views computers as tools easily separable from their human users.

### Determining InfoSec goals for the whole biocybernetic user-device system

At a minimum, such analyses suggest that the CIA Triad might better be interpreted as requiring the confidentiality, integrity, and availability of information contained within the hybrid user-device system *as a whole*, rather than simply within its neuroprosthetic component. InfoSec mechanisms designed to protect information contained in a neuroprosthesis at all costs (e.g., by “failing closed” in case of a hardware problem) may endanger the safety and agency of its human host, while InfoSec practices that focus only on securing the biological elements of a user's organism may result in weak device security, thereby allowing devices to be compromised in ways that negatively impact their users. Such extremes of “subsystem optimization” can be prevented by keeping in mind the goal of optimizing InfoSec for the larger biocybernetic system formed through the coaction of a neuroprosthesis and its host. Because that whole *includes* a sapient human being possessing a unique legal and moral status (Wallach and Allen, 2008; Gunkel, 2012; Sandberg, 2014), technical issues become intertwined with complex social and philosophical questions.

At a deeper level, though, such analyses raise the question of whether a CIA Triad formulated decades ago for securing rudimentary electronic computers offers a viable starting point for developing robust InfoSec schemas for a human-computer whole. Indeed, it appears unlikely that human beings would spontaneously identify “confidentiality,” “integrity,” and “availability” of information as the most critical considerations for technologies that have such direct impacts on their own long-term psychological, physical, and social wellbeing (Denning et al., 2010; Bonaci et al., 2015b).

## From disconnected goals to a coherent axiology of InfoSec values for futuristic neuroprostheses

Futuristic neuroprostheses create many distinct layers of InfoSec concerns: for example, an immersive neuroprosthetic VR system that allows its user to stroll through a “virtual city” might not only threaten the integrity of the user's cerebral information system at the basic biological level by physically damaging his or her neurons; it could also distort that information system's contents at a higher semantic level by, for example, allowing the user to read “virtual newspapers” that contain blatantly false information.

**Figure 1:**
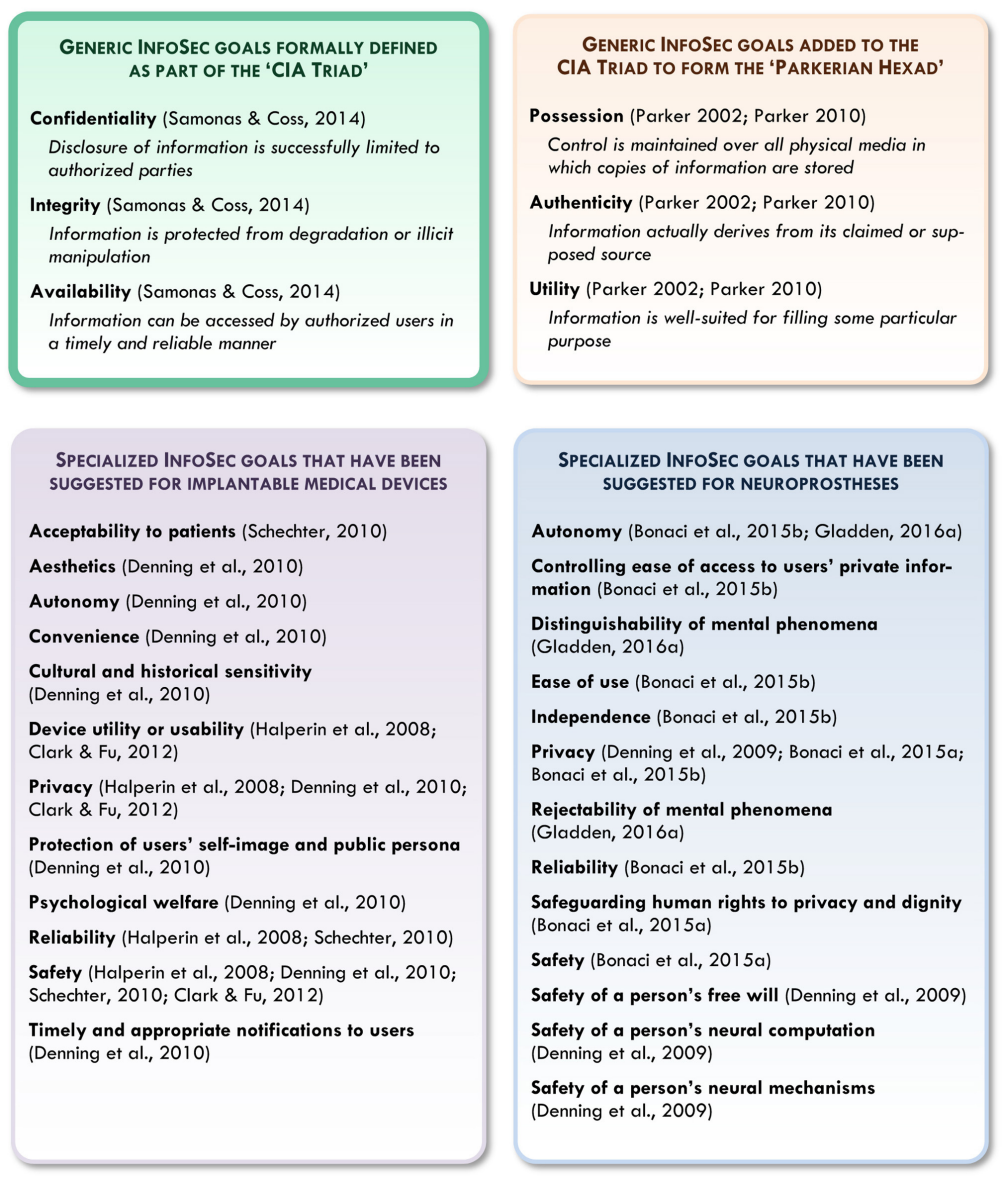
A comparison of the generalized InfoSec goals formally defined in the CIA Triad and Parkerian Hexad with more specialized goals that have been suggested for implantable medical devices and neuroprostheses (including summaries or paraphrases of InfoSec goals that are discussed but not explicitly defined in their respective texts).

With such challenges in mind, researchers have begun to informally identify a range of possible InfoSec goals relevant for futuristic neuroprostheses. As indicated in Figure 1, specialized InfoSec goals suggested for IMDs and their users include *device reliability; utility or usability; convenience; aesthetics; sensitivity to cultural and historical associations*; *acceptability to patients*; *adequate notification to users*; and *protection of users' safety, privacy, autonomy, psychological welfare, self-image, and public persona* (Halperin et al., 2008; Denning et al., 2010; Schechter, 2010; Clark and Fu, 2012). In the specific case of neuroprostheses, suggested InfoSec goals include *device reliability; ease of use; safety* (including *safety for users' neural mechanisms and computational processes*); the *distinguishability and rejectability of mental phenomena by users*; *protection of users' independence, free will, and human rights to privacy and dignity;* and *the autonomy of users and user-device systems* (Denning et al., 2009; Bonaci et al., 2015a,b; Gladden, 2016a).

While some of these goals are directly ethical or legal in nature, others are primarily technical and technological—and still others (e.g., relating to convenience, cultural appropriateness, aesthetics, and a user's public persona) represent different sorts of aims that cannot be reduced simply to matters of law, ethics, or technical effectiveness. This suggests that InfoSec for futuristic neuroprostheses can be usefully analyzed through the lens of *axiology*, the philosophical investigation of values. Axiology encompasses not only ethics (with its consideration of actions that are right or wrong) but also aesthetics (with its investigation of goods like truth, harmony, and functionality) and the study of values associated with other types of goods.

Axiology allows us to identify, classify, understand, and prioritize goods in different ways. For example, some suggested InfoSec goals (like those relating to usability) might be understood as *instrumental goods*, which are valued because they allow us to achieve another desired end; other InfoSec goals (like those relating to safety and dignity) could be understood as intrinsic goods, which are valued in themselves because they are considered inherently worthwhile (Weber, 1978). InfoSec goals for neuroprostheses can also be classified according to whether they relate to ethical, aesthetic, physical (e.g., health-related), metaphysical, technological, psychological, historical, religious, economic, or social values (Hartman, 2011).

The information stored or processed by a neuroprosthesis reflects a complex tangle of such goods and values. For example, rich data regarding the functioning of a user's brain may possess not only *instrumental economic value* (e.g., if exploited by a device manufacturer) but also *intrinsic aesthetic value* (e.g., insofar as it reflects intricate biological patterns and elegant physical laws that manifest a certain natural beauty). Even superficial cosmetic aspects of a device can disclose significant information regarding the ethical, aesthetic, physical, technological, psychological, religious, economic, and social values held by its designer and user.

**Figure 2:**
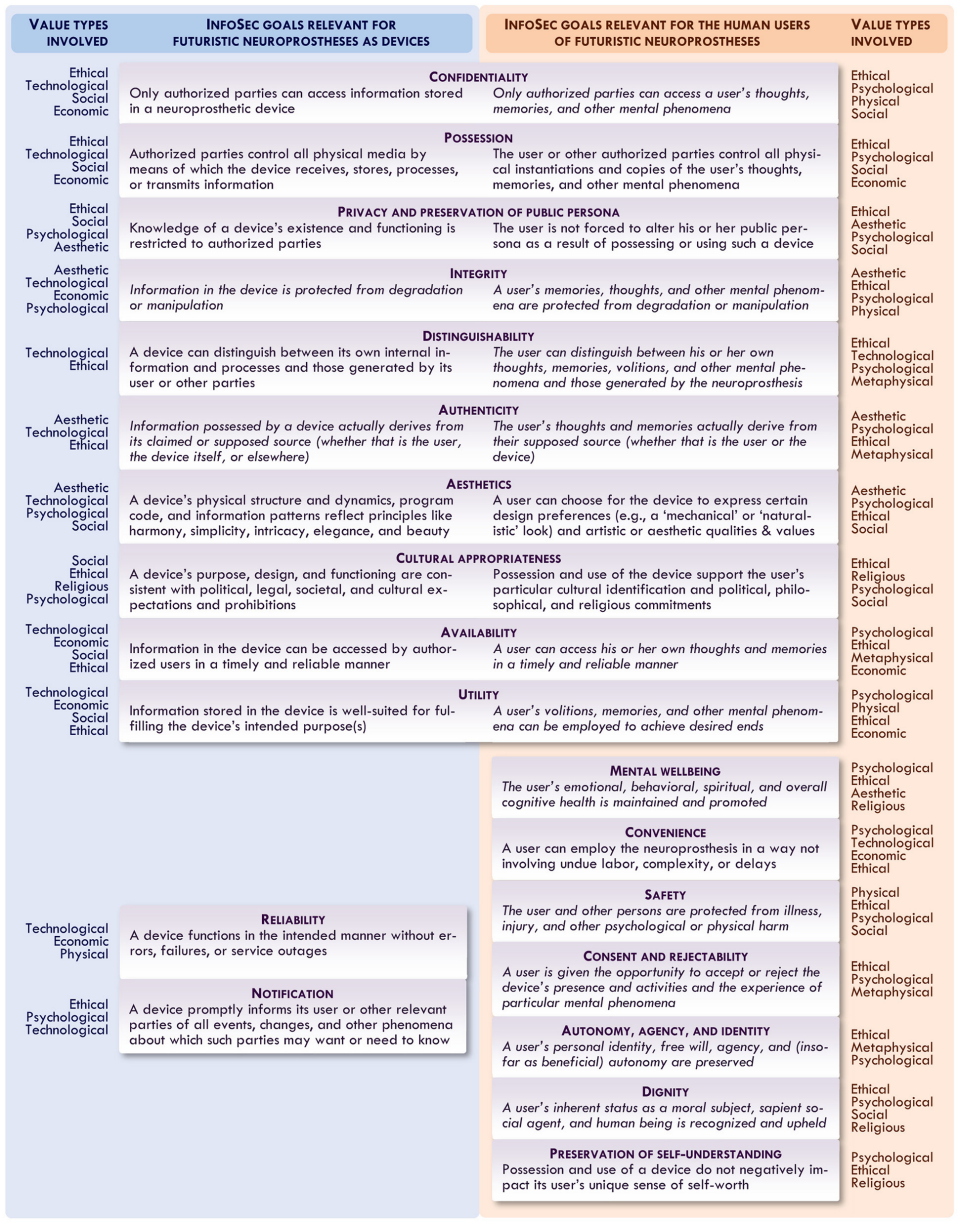
A proposed axiological framework for analyzing InfoSec goals for futuristic neuroprostheses that are relevant particularly for devices (left) and their users (right); in the margins are noted values (ethical, psychological, physical, etc.) especially associated with a given InfoSec goal. Goals described in italics are those more likely to be recognized as intrinsic goods from some axiological perspectives.

While there is much debate in the field of axiology surrounding such issues, there is broad agreement that, for example, in case of conflict, an *instrumental economic or technological good* (like that of ensuring a device's reliability or ease of use) should be given lower priority than an *intrinsic moral good* (like that of protecting users' safety or free will). As Figure 2 suggests, superficially similar InfoSec goals may represent very different types of goods: safeguarding the availability of information in a neuroprosthesis may be an instrumental technological good, while safeguarding the availability of information in its user's mind might be an intrinsic moral and psychological good.

The exact nature of a neuroprosthesis also heavily influences which InfoSec issues and values will be relevant: for example, a noninvasive wearable visual neuroprosthesis, retinal implant, and visuocortical implant relate to the mind and body in different ways and raise very different issues. Many insights might be gained from the substantial existing body of axiological research regarding futuristic autonomous robots—especially since many futuristic neuroprostheses meet the definition of a specialized type of “robot” (Murphy, 2000; Bekey, 2005; Wallach and Allen, 2008; Gunkel, 2012).

## Conclusion

In this text we have argued that as long as increasingly outdated instrumental schemas like the CIA Triad remain the default or “best” definition of InfoSec goals available to neuroprosthetics researchers, it will be difficult to develop InfoSec regimes for futuristic neuroprostheses that adequately address the complex issues they raise regarding human agency and human-machine integration. It is hoped that by formulating more robust axiological InfoSec frameworks of the sort sketched above—which look beyond instrumental approaches to consider the relationship of “information” and “information systems” to a wide range of values and goods—futuristic neuroprostheses and their users can be protected against dangers including not only conventional data theft or financial loss but also threats to the essential dynamics of memory, consciousness, conscience, and autonomy that lie at the heart of what makes us human.

## Author contributions

The author confirms being the sole contributor of this work and approved it for publication.

### Conflict of interest statement

The author declares that the research was conducted in the absence of any commercial or financial relationships that could be construed as a potential conflict of interest.
